# Prognostic significance and influencing factors of lipomatous metaplasia in patients after myocardial infarction

**DOI:** 10.1186/s13244-025-02152-w

**Published:** 2025-12-08

**Authors:** Yan Chen, Xuelian Gao, Weibo Li, Nan Zhang, Yue Ren, Yifeng Gao, Zhen Zhou, Jiayi Liu, Zhaoying Wen, Lei Xu

**Affiliations:** 1 Department of Radiology, Beijing Anzhen Hospital, Capital Medical University, Beijing, China; 2 Department of Radiology, Nanchong Central Hospital, Nanchong, China

**Keywords:** Adipose tissue, Myocardial infarction, Prognosis, Computed tomography, Magnetic resonance imaging

## Abstract

**Objectives:**

To investigate the prognostic value of lipomatous metaplasia (LM) in patients after myocardial infarction (MI) and to explore potential influencing factors of LM.

**Materials and methods:**

A total of 1702 (mean age 59.3 ± 10.27 years, 86.08% men) patients with a history of MI who underwent coronary CT angiography (CCTA) examinations were retrospectively enrolled. The clinical endpoints were major adverse cardiovascular events (MACE). A subgroup of 240 patients who underwent CCTA and cardiac magnetic resonance (CMR) examinations within a 14-day interval was analyzed to compare the prognostic values of LM and CMR parameters and to explore influencing factors of LM.

**Results:**

MACE occurred in 395 (23.21%) patients during a median follow-up of 45.5 months. In the entire cohort, the prevalence of LM was 46.71% on CCTA; in the subgroup, it was 51.25% on CCTA and 21.67% on CMR. LM remained a significant outcome predictor (hazard ratio (HR) 1.39, 95% confidence interval (CI) 1.12–1.73; *p* = 0.002) in the multivariable model. In subgroup analysis, LM on CCTA (HR 1.83, 95% CI 1.09–3.08; *p* = 0.023) was a stronger outcome predictor than all CMR parameters. Revascularization history (odds ratio (OR) 2.833, *p* = 0.006), number of diseased coronary arteries (CA) (OR 0.556, *p* = 0.006) and infarct size (OR 1.094, *p* = 0.003) remained associated with LM in the multivariable model.

**Conclusion:**

LM was a significant outcome predictor in patients after MI and was stronger than CMR functional parameters and infarct size. Revascularization, infarct size and fewer diseased CA may be associated with LM development.

**Critical relevance statement:**

Lipomatous metaplasia (LM) was a common complication following myocardial infarction (MI) that increased with infarct age, identifying LM and integration of LM assessment into risk stratification models for post-MI patients may be important for clinical strategy decisions.

**Key Points:**

- Lipomatous metaplasia was a common complication that increased with infarct age.
- Lipomatous metaplasia was a significant outcome predictor in patients after myocardial infarction, stronger than CMR functional parameters and infarct size.
- Revascularization procedure, infarct size and fewer number of diseased coronary arteries were associated with the presence of lipomatous metaplasia.

**Graphical Abstract:**

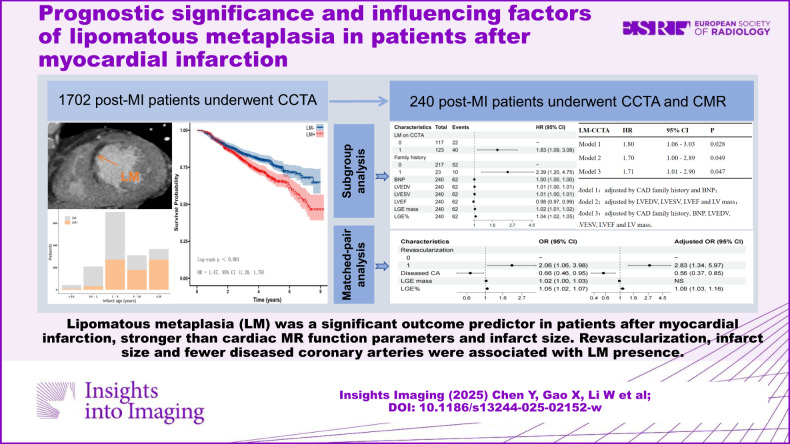

## Introduction

Lipomatous metaplasia (LM) refers to the replacement of the myocardial scar with adipose tissue after myocardial infarction (MI) [1]. It is a common but inadequately understood form of ventricular remolding after MI that has only been described in recent decades [1, 2]. Studies have reported that LM, rather than myocardial scar, is the substrate for ventricular tachycardia (VT) within the postinfarct myocardium [3, 4]. However, the prognostic value of LM after MI has not been sufficiently examined in large cohorts.

Coronary CT angiography (CCTA) is the first-line imaging modality for anatomic and functional assessment of the coronary artery in patients with ischemic cardiomyopathy [5]. Furthermore, this procedure is used to detect myocardial LM noninvasively via attenuation threshold owing to the advantage of submillimeter spatial resolution [6]. Cardiac magnetic resonance (CMR) imaging is the “one-stop” method to evaluate cardiac function and myocardial viability [5]. Conventional functional parameters and late gadolinium enhancement (LGE) image-based infarct size are considered reliable outcome predictors in MI [7–9].

Identifying the predisposing factors for LM may be important for risk stratification and therapeutic strategy guidance in patients after MI, but the results from previous studies are uncertain and controversial [1–3, 10].

Therefore, this study aimed to investigate the prognostic value of LM detected on CCTA images in a large cohort, compare it with CMR functional parameters and infarct size in a subgroup of patients who had undergone CCTA and CMR examinations within a 14-day interval, and explore the potential influencing factors of LM.

## Materials and methods

### Study population

This retrospective study was approved by the ethics committee of Beijing Anzhen Hospital (No. 2021164X). All examinations were performed for clinical reasons, and the requirement for written informed consent was waived.

**Fig. 1:**
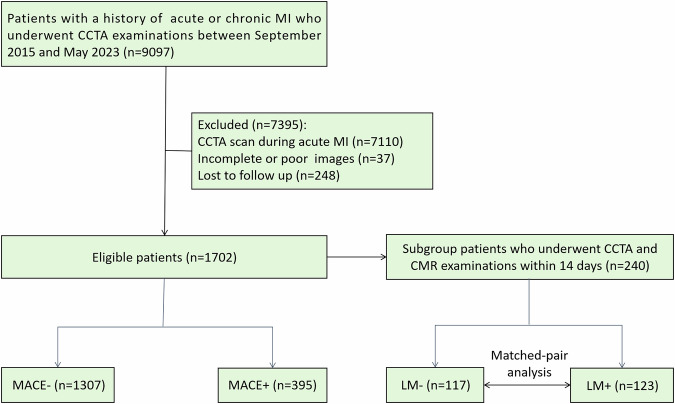
Flowchart of patient selection. MI, myocardial infarction; CCTA, coronary computed tomography angiography; CMR, cardiovascular magnetic resonance; MACE, major adverse cardiac events; LM, lipomatous metaplasia

The medical records of 9097 patients with a history of acute or chronic MI who had undergone CCTA examinations between September 2015 and May 2023 at our institution were retrospectively reviewed. Acute MI was diagnosed based on the presence of acute myocardial injury, as inferred from abnormal cardiac biomarkers in the setting of evidence of acute myocardial ischemia (requiring at least one of the following: ① symptoms of acute myocardial ischemia; ② new ischemic ECG changes; ③ development of pathological Q waves; ④ imaging evidence of new loss of viable myocardium or new regional wall motion abnormality in a pattern consistent with an ischemic etiology) [11]. Chronic MI was diagnosed based on clinical symptoms, pathological Q waves on electrocardiogram (ECG), and characteristic CMR patterns [12]. Patients who underwent CCTA examination during acute MI were excluded, as well as those with incomplete or poor CCTA or CMR images. Patients lost to follow-up were also excluded. A subgroup of patients who had undergone CCTA and CMR examinations within a 14-day interval was analyzed to compare the prognostic values of LM and CMR functional parameters and infarct size. Furthermore, the potential influencing factors of LM were determined by performing a matched-pair analysis adjusted for age, sex, body mass index (BMI), and infarct age. The matched-pair analysis was performed only for a subgroup of patients who underwent CMR. Laboratory tests were performed within 1 week before CCTA examinations. The flowchart detailing the process of patient selection is presented in Fig. 1.

### CCTA and CMR protocols

CCTA examinations were performed using four different multidetector scanners. 50 to 60 mL of the contrast agent Ultravist iopromide (370 mg/mL, Bayer) was injected into the antecubital vein at a speed of 4.0 to 4.5 mL/s, followed by 30 to 35 mL of 0.9% saline solution with the same injection speed. The scan parameters were set according to the Society of Cardiovascular Computed Tomography guidelines [13]. CMR examinations were performed using three different 3.0-T systems. Cine images were acquired before contrast injection. The gadolinium-based contrast agent (Magnevist, Bayer Healthcare) was injected into the antecubital vein at a dose of 0.2 mmol/kg, followed by a 20 mL of 0.9% saline solution. About 10 min later, LGE images were acquired. The scan parameters were set according to standardized CMR protocols [14]. The detailed scanning parameters of CCTA and CMR examinations are elaborated in the Supplementary Data.

### CCTA and CMR image analysis

Two radiologists (Y.C. and N.Z., with 5 and 10 years of experience, respectively, in cardiovascular image interpretation) independently analyzed all CCTA and CMR images. They were both blinded to the clinical data. In case of a difference of opinion, an agreement was reached via discussion with another radiologist (L.X., with 20 years of experience). A random cohort of 200 patients was reanalyzed after a 30-day interval by Y.C. to assess intraobserver agreement.

**Fig. 2:**
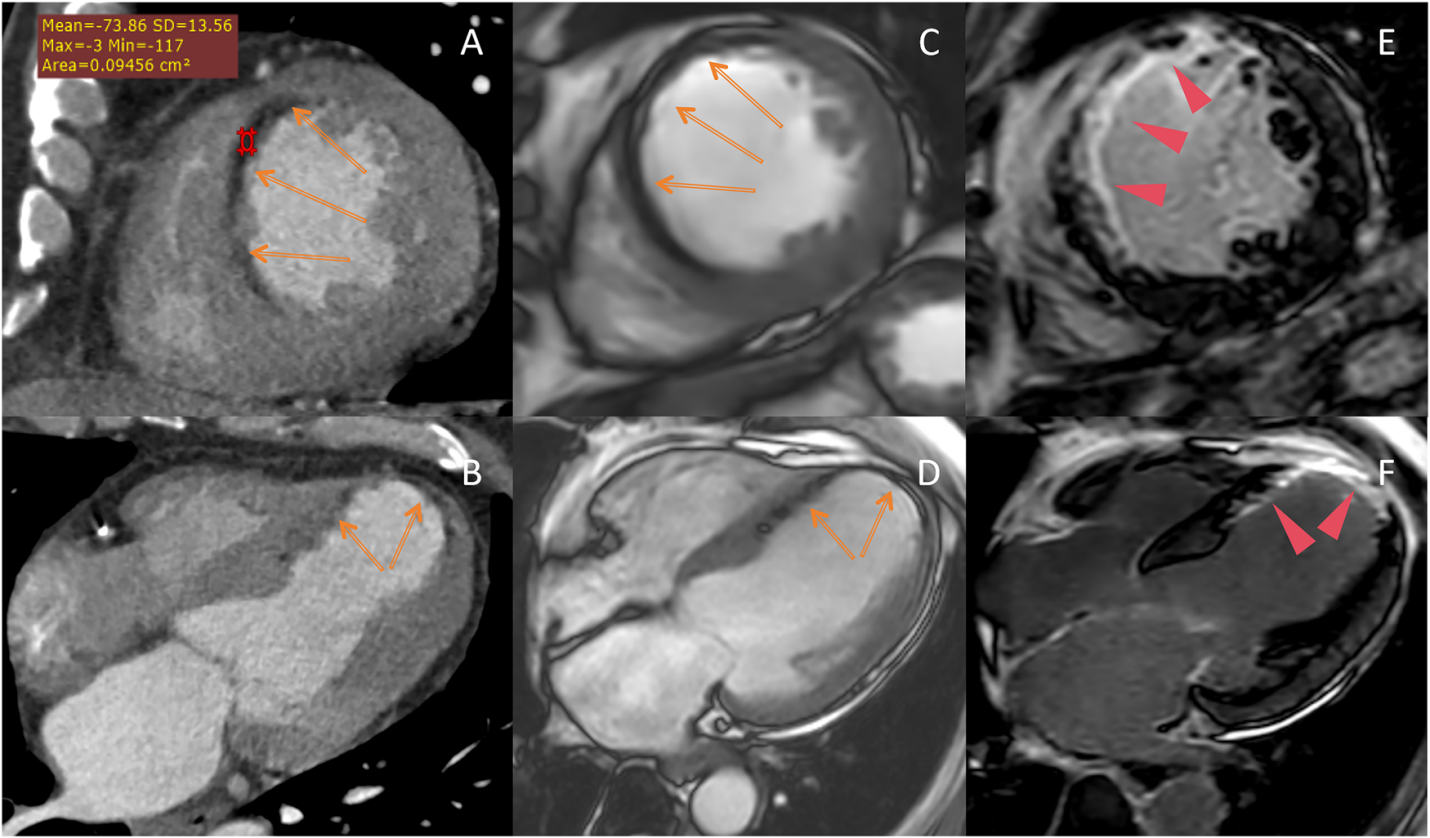
Lipomatous metaplasia in a 63-year-old patient with a history of anteroseptal wall infarction (infarct age 1 year). CCTA images (**A**, **B**) showed subendocardial fat (−73.86 HU) in the anteroseptal wall (arrows). CMR cine images (**C**, **D**) revealed the presence of subendocardial fat (arrows) within the myocardial scar (arrowheads) on LGE images (**E**, **F**), and the size of the subendocardial fat was smaller than that of the myocardial scar. **A**, **C**, **E**: short axis plane; **B**, **D**, **F**: 4-chamber long axis plane. LGE, late gadolinium enhancement

Axial and multiplanar reformation CCTA images and CMR cine and LGE images were analyzed on the PACS workstation to determine the presence of LM within the left ventricular (LV) myocardium. LM was assessed visually using multiplanar reconstruction on CCTA and only visually on CMR cine images. The criterion for LM presence on CCTA images was a minimal continuous area of > 1 mm^2^, with attenuation between −180 and 0 HU within the LV myocardium perfused by the infarct-related artery [6] (Fig. 2A, B). Fat within the right ventricular wall or adipose tissue of LV myocardium outside the perfusion territory of the culprit vessel was not regarded as LM after MI. The criterion for LM presence on steady-state free precession (SSFP) sequences was a central high signal intensity surrounded by a low signal intensity band owing to the cancellation of the fat and water intravoxel signals, known as chemical shift artifacts [15] (Fig. 2C–F).

Coronary artery involvement was evaluated using invasive coronary angiography or CCTA images. A diseased coronary artery is one with luminal stenosis > 50%. Stenosis of the left main artery, left anterior descending artery, right coronary artery, and left circumflex artery was evaluated; stenosis of branches was included in 3 main vessels, which means that the number of diseased coronary arteries ranged from 0 to 4.

LV function and myocardial scar were assessed based on CMR cine and LGE images using the Cvi42 software (version 5.2.0, Circle). Myocardial scar was defined as the region with a signal intensity five standard deviations (SDs) greater than the mean signal of the remote myocardial region of interest on the LGE image [16] and expressed as myocardial scar mass (LGE mass) and proportion (LGE mass as a percentage of LV mass, LGE%, which represents infarct size).

### Clinical endpoints

All patients were followed up via chart review or telephonic interviews. Experienced staff at our institution’s follow-up center conducted the follow-up and recorded the results in the clinical research database. The clinical endpoints were MACE, defined as all-cause death, heart transplantation, myocardial reinfarction, heart failure or unstable angina hospitalization, sustained ventricular tachycardia, ventricular fibrillation, and stroke [17]. The follow-up period was from the time of the CCTA scan to the date of follow-up or event. Patients lost to follow-up (248 out of 1950, 12.72%) were excluded from further analysis. Finally, a total of 1702 patients (mean age 59.3 ± 10.27 years, 86.08% men) were included in the final analysis.

### Statistical analysis

All statistical analyses were performed using the R software (version 4.2.2). Normally distributed continuous variables were expressed as mean ± SD and compared using an independent-samples *t*-test. Non-normally distributed continuous variables were expressed as median with interquartile range (IQR) and compared using the Mann–Whitney U test. Categorical variables were expressed as counts and percentages and compared using the chi-square test or Fisher’s exact test. In the case of missing data, mean values were imputed for continuous variables when the frequency was less than 5%; otherwise, cases with missing data were omitted. Reproducibility analysis was completed using intraclass correlation coefficients (ICCs). Cox proportional hazards regression analysis and Kaplan–Meier survival analysis were used to determine the prognostic value of LM. Logistic regression was used to analyze the association between LM presence and other parameters in the matched-pair analysis. All tests were two-tailed, and a *p*-value of < 0.05 was considered statistically significant.

## Results

### Follow-up

In the entire study cohort, during a median follow-up of 45.5 [IQR 28.0–59.7] months, 395 out of 1702 patients (23.21%) experienced MACE, which included all-cause death (55, 3.23%), heart transplantation (3, 0.18%), myocardial reinfarction (49, 2.88%), heart failure hospitalization (87, 5.11%), unstable angina hospitalization (180, 10.58%), sustained ventricular tachycardia (8, 0.47%), ventricular fibrillation (1, 0.06%), and stroke (12, 0.71%).

In the subgroup cohort, the median follow-up time was 46.8 [IQR 21.6–71.0] months, during which 62 out of 240 patients (25.83%) experienced MACE. The events included all-cause death (15, 6.25%), myocardial reinfarction (4, 1.67%), heart failure hospitalization (21, 8.75%), unstable angina hospitalization (21, 8.75%), and sustained ventricular tachycardia (1, 0.42%).

### Patient characteristics

**Fig. 3:**
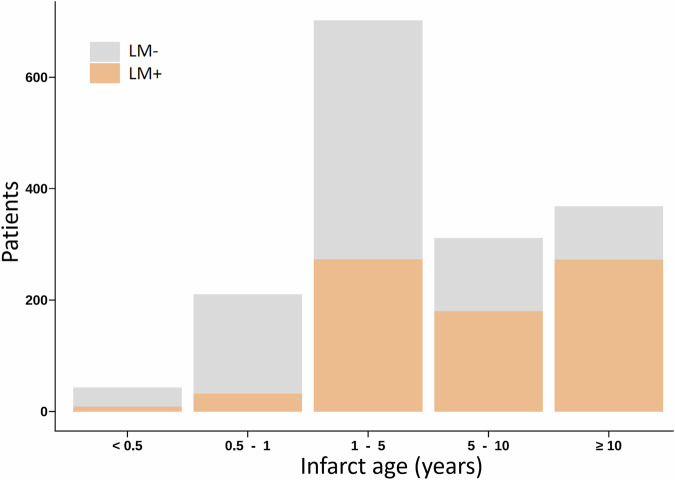
Relationship between LM presence and infarct age in patients after MI. The incidence of LM in post-MI patients increased with infarct age. LM was observed in 9 cases (2.09%) within 6 months after MI. The prevalence of LM within 1 year after MI was relatively low, with only approximately 16.21%. The proportion of patients developing LM (LM+) remained lower than those without LM (LM−) within the first 5 years post-infarction. However, this proportion increased markedly after 5 years, and the prevalence of LM reached 66.57% in infarcts older than 5 years

Patient characteristics are summarized in Table 1. The median infarct age was 3.0 [IQR 1.0–9.0] years. In the entire study cohort, LM within the LV myocardium was detected in 795 out of 1702 patients (46.71%) based on CCTA images. The incidence of LM in post-MI patients increased with infarct age. LM was observed in 9 cases (2.09%) within 6 months after MI. The prevalence of LM within 1 year after MI was relatively low, with only approximately 16.21%. The proportion of patients developing LM (LM+) remained lower than those without LM (LM−) within the first 5 years post-infarction. However, this proportion increased markedly after 5 years, and the prevalence of LM reached 66.57% in infarcts older than 5 years. The relationship between LM presence and infarct age is illustrated in Fig. 3. Notably, the infarct age of LM+ patients was significantly higher than that of LM− patients (6.0 [IQR 2.0–12.0] years vs. 2.0 [IQR 1.0–5.0] years; *p* < 0.001).

**Table 1:** Patient characteristics

	All patients (*n* = 1702)	LM on CCTA (−) (*n* = 907)	LM on CCTA (+) (*n* = 795)	*p*-value
Age, years	59.3 ± 10.27	58.7 ± 10.54	59.9 ± 9.92	**0.013**
Sex, male, *n* (%)	1465 (86.08%)	766 (84.45%)	699 (87.92%)	**0.039**
Body mass index, kg/m^2^	26.02 ± 3.28	25.93 ± 3.44	26.13 ± 3.09	0.216
Infarct age (years)	3.0 (1.0, 9.0)	2.0 (1.0, 5.0)	6.0 (2.0, 12.0)	**< 0.001**
< 0.5	43 (2.63%)	34 (3.92%)	9 (1.17%)	
0.5–1.0	210 (12.85%)	178 (20.51%)	32 (4.18%)	
1.0–5.0	702 (42.96%)	429 (49.42%)	273 (35.64%)	
5.0–10.0	311 (19.03%)	131 (15.09%)	180 (23.50%)	
≥ 10.0	368 (22.52%)	96 (11.06%)	272 (35.51%)	
Hypertension, *n* (%)	1058 (62.16%)	592 (65.27%)	466 (58.62%)	**0.005**
Diabetes mellitus, *n* (%)	637 (37.43%)	329 (36.27%)	308 (38.74%)	0.294
Hyperlipoidemia, *n* (%)	953 (55.99%)	506 (55.79%)	447 (56.23%)	0.856
Smoking, *n* (%)	1086 (63.81%)	567 (62.51%)	519 (65.28%)	0.236
Family history, *n* (%)	108 (6.35%)	64 (7.06%)	44 (5.53%)	0.199
Revascularization history, *n* (%)	1315 (77.26%)	657 (72.44%)	658 (82.77%)	**< 0.001**
PCI	981 (74.60%)	492 (74.88%)	489 (74.32%)	
CABG	184 (13.99%)	93 (14.15%)	91 (13.83%)	
PCI + CABG	150 (11.41%)	72 (10.96%)	78 (11.85%)	
Number of diseased coronary arteries				0.535
No significant vessel stenosis, *n* (%)	3 (0.18%)	2 (0.22%)	1 (0.13%)	
1-vessel disease, *n* (%)	431 (25.32%)	239 (26.35%)	192 (24.15%)	
2-vessel disease, *n* (%)	417 (24.50%)	211 (23.26%)	206 (25.91%)	
Multi-vessel disease (≥ 3), *n* (%)	851 (50.00%)	455 (50.17%)	396 (49.81%)	
Laboratory results				
cTNI, ng/mL	0.000 (0.000, 0.010)	0.000 (0.000, 0.010)	0.000 (0.000, 0.010)	0.759
CK-MB, ng/mL	1.70 (1.20, 2.60)	1.70 (1.10, 2.60)	1.70 (1.20, 2.50)	0.876
BNP, pg/mL	80.00 (34.00, 190.01)	83.00 (31.00, 190.01)	78.00 (37.00, 190.01)	0.896
hs-CRP, mg/L	1.01 (0.48, 2.96)	1.03 (0.51, 3.00)	0.98 (0.45, 2.87)	0.175

*LM* lipomatous metaplasia, *CCTA* coronary computed tomography angiography, *PCI* percutaneous coronary intervention, *CABG* coronary artery bypass grafting, *cTnI* cardiac troponin I, *CK-MB* creatine kinase, *BNP* B-type natriuretic peptide, *hs-CRP* high-sensitivity C-reactive protein. Statistically significant *p*-values are in bold

The agreement was excellent for LM on CCTA and good for LM on CMR. For LM on CCTA, the intra- and inter-observer ICCs were 0.978 and 0.887. For LM on CMR, the intra- and inter-observer ICCs were 0.947 and 0.821 (see Table S1).

### Prognostic value of LM

**Fig. 4:**
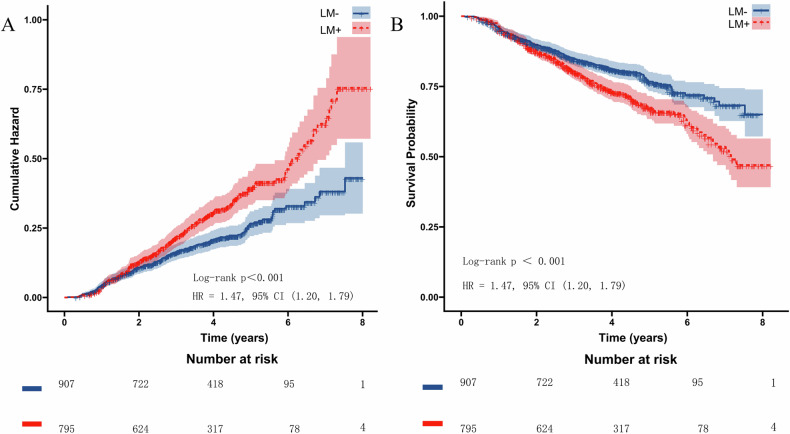
Cumulative hazard (**A**) and survival (**B**) curves for the entire study cohort, stratified by LM presence on CCTA. The presence of LM was significantly associated with a higher cumulative hazard and a lower probability of survival in post-MI patients

In patients with MACE, the proportion of LM+ patients was higher (55.19% vs. 44.15%, *p* < 0.001), the infarct age was older (4.0 [IQR 1.3–10.0] years vs. 3.0 [IQR 1.0–8.0] years; *p* = 0.004) (see Table S2 in the Supplementary Data). Table 2 presents the results of univariable and multivariable Cox regression analysis in the entire study cohort. In the univariable analysis, LM on CCTA, infarct age, diabetes mellitus, coronary artery disease (CAD) family history, revascularization history, and B-type natriuretic peptide (BNP) level were significantly linked to MACE occurrence. These parameters were subsequently entered into a multivariable model, and the results showed that LM remained significantly associated with MACE when adjusted for other significant clinical factors (hazard ratio (HR) 1.39, 95% CI 1.21‒1.730, *p* = 0.002). Kaplan–Meier plots of MACE stratified by LM presence are depicted in Fig. 4.

**Table 2:** Univariable and multivariable Cox regression results for MACE association in the entire study cohort

	Univariable analysis		Multivariable analysis	
	Hazard ratio (95% CI)	*p*-value	Hazard ratio (95% CI)	*p*-value
LM on CCTA, *n* (%)	1.47 (1.20, 1.79)	**< 0.001**	1.39 (1.21, 1.73)	**0.002**
Age, years	1.01 (1.00, 1.02)	0.100		
Sex, male, *n* (%)	1.07 (0.81, 1.42)	0.616		
Body mass index, kg/m^2^	0.99 (0.96, 1.02)	0.514		
Infarct age (years)	1.02 (1.01, 1.04)	**0.005**	1.01 (0.99, 1.03)	0.190
Hypertension, *n* (%)	1.11 (0.91, 1.37)	0.307		
Diabetes mellitus, *n* (%)	1.26 (1.03, 1.54)	**0.024**	1.20 (0.97, 1.47)	0.086
Hyperlipoidemia, *n* (%)	1.14 (0.93, 1.39)	0.200		
Smoking, *n* (%)	1.11 (0.90, 1.37)	0.324		
Family history, *n* (%)	1.50 (1.06, 2.13)	**0.024**	1.52 (1.06, 2.18)	**0.022**
Revascularization history, *n* (%)	1.29 (1.01, 1.65)	**0.044**	1.23 (0.94, 1.61)	0.134
Number of diseased coronary arteries	1.00 (0.90, 1.11)	0.966		
Laboratory results				
cTNI, ng/mL	1.00 (0.99, 1.00)	0.820		
CK-MB, ng/mL	1.00 (0.99, 1.01)	0.996		
BNP, pg/mL	1.00 (1.00, 1.00)	**< 0.001**	1.00 (1.00, 1.00)	**< 0.001**
hs-CRP, mg/L	1.01 (0.99, 1.02)	0.266		

*95% CI* 95% confidence interval, *LM* lipomatous metaplasia, *CCTA* coronary computed tomography angiography, *cTnI* cardiac troponin I, *CK-MB* creatine kinase, *BNP* B-type natriuretic peptide, *hs-CRP* high-sensitivity C-reactive protein. Statistically significant *p*-values are in bold

### Subgroup analysis

A cohort of 240 patients (mean age 56.8 ± 10.90 years, 87.08% men) was included in the subgroup analysis. The median infarct age of the subgroup patients was 1.5 [IQR 0.5–5.0] years. Patient characteristics of the subgroup cohort are furnished in the Supplementary Data (Table S3).

LM was observed in 123 (51.25%) patients based on CCTA images, but only in 52 (21.67%) patients based on CMR SSFP sequences. It was observed that in all 52 patients who presented LM on CMR SSFP sequences, fat attenuation was confirmed on CCTA images. Based on CMR cine and LGE images, LM was found to be present only within the LGE hyperintense area (i.e., myocardial scar), but the size of LM was always smaller than that of the myocardial scar. For 71 patients, the regions exhibiting LM on CCTA images appeared as scattered patches, with non-fatty regions interspersed among multiple clusters of fat. In these cases, LM was not detectable on CMR SSFP sequences. LM presence was more frequently observed in patients with MACE than in those without MACE; however, the difference was significant only when LM was determined on CCTA images (64.52% vs. 46.63%, *p* = 0.015).

**Fig. 5:**
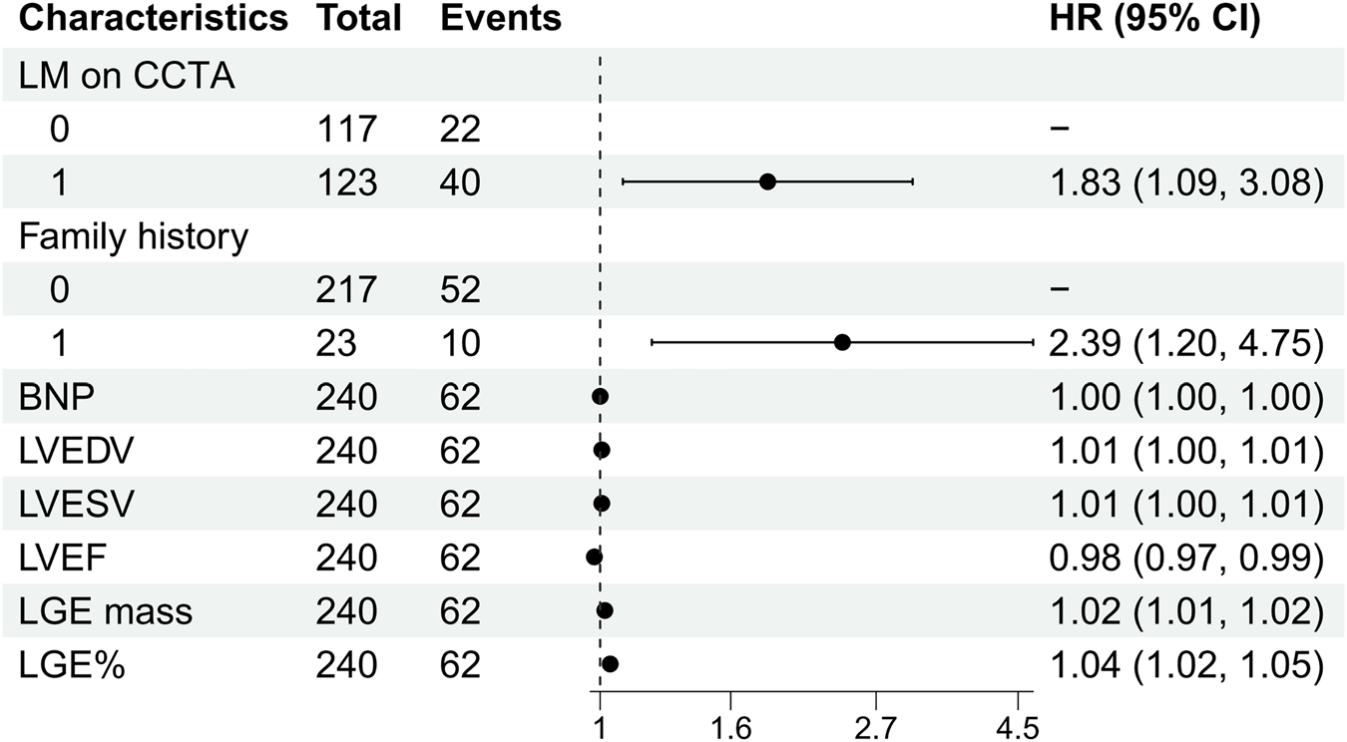
Forest plot of hazard ratios for associations with MACE in the subgroup cohort. LM presence based on CCTA was a stronger outcome predictor than all CMR functional parameters and infarct size

Univariable and multivariable Cox regression results of the association of LM presence and clinical and CMR parameters with MACE are displayed in the Supplementary Data (Table S4). LM on CCTA was significantly associated with MACE, but LM on CMR was not. The prognostic value of LM on CCTA (HR 1.83, 95% CI 1.09–3.08, *p* = 0.023) was stronger than that of all significant CMR parameters. The forest plot of HRs of significant factors is presented in Fig. 5. LM on CCTA remained significantly associated with MACE when adjusted for family history, BNP (model 1, HR 1.80, 95% CI 1.06–3.03, *p* = 0.028); LVEDV, LVESV, LVEF, LV mass (model 2, HR 1.70, 95% CI 1.00–2.89, *p* = 0.049); and combined model 1 and model 2 (model 3, HR 1.71, 95% CI 1.00–2.90, *p* = 0.047), as presented in Table 3.

**Table 3:** Multivariable Cox regression models of LM on CCTA with MACE in subgroup analysis

LM on CCTA	Hazard ratio	95% CI	*p*-value
Model 1	1.80	1.06–3.03	0.028
Model 2	1.70	1.00–2.89	0.049
Model 3	1.71	1.01–2.90	0.047

Model 1 adjusted for: family history, BNP. Model 2 adjusted for: LVEDV, LVESV, LVEF, LV mass. Model 3 adjusted for: family history, BNP, LVEDV, LVESV, LVEF, LV mass

*LV* left ventricular, *EDV* left ventricular end diastolic volume, *LVESV* left ventricular end systolic volume, *LVSV* left ventricular stroke volume, *LVEF* left ventricular ejection fraction

### Matched-pair analysis

In the matched-pair analysis, more patients received revascularization in the LM+ group than in the LM− group (60.27% vs. 42.47%, *p* = 0.031). LGE% remained higher in the LM+ group than in the LM− group (25.52% [IQR 17.02%–34.71%]; vs. 17.38% [IQR 9.85%–26.73%]; *p* < 0.001) (Table 4).

**Table 4:** Matched-pair analysis grouped by LM presence on CCTA

	All patients (*n* = 146)	LM on CCTA (−) (*n* = 73)	LM on CCTA (+) (*n* = 73)	*p*-value
Age, years	58.2 ± 10.43	58.9 ± 10.63	57.5 ± 10.25	0.425
Sex, male, *n* (%)	128 (87.67%)	64 (87.67%)	64 (87.67%)	1.000
Body mass index, kg/m^2^	25.54 ± 3.37	25.17 ± 3.32	25.92 ± 3.39	0.180
Infarct age (years)	1.2 (0.6, 3.4)	1.0 (0.5, 4.0)	1.5 (1.0, 3.0)	0.156
Hypertension, *n* (%)	91 (62.33%)	48 (65.75%)	43 (58.90%)	0.393
Diabetes mellitus, *n* (%)	67 (45.89%)	37 (50.68%)	30 (41.10%)	0.245
Hyperlipoidemia, *n* (%)	72 (49.32%)	35 (47.95%)	37 (50.68%)	0.714
Smoking, *n* (%)	91 (62.33%)	48 (65.75%)	43 (58.90%)	0.393
Family history, *n* (%)	11 (7.53%)	6 (8.22%)	5 (6.85%)	0.754
Revascularization history, *n* (%)	75 (51.37%)	31 (42.47%)	44 (60.27%)	**0.031**
Number of diseased coronary arteries				0.135
1-vessel disease, *n* (%)	37 (25.34%)	14 (19.18%)	23 (31.51%)	
2-vessel disease, *n* (%)	29 (19.86%)	12 (16.44%)	17 (23.29%)	
Multi-vessel disease (≥ 3), *n* (%)	80 (54.79%)	47 (64.38%)	33 (45.21%)	
Laboratory results				
cTNI, ng/mL	0.010 (0.000, 0.045)	0.010 (0.000, 0.030)	0.010 (0.000, 0.068)	0.235
CK-MB, ng/mL	2.20 (1.50, 3.30)	2.10 (1.40, 3.20)	2.40 (1.50, 4.18)	0.327
BNP, pg/mL	85.00 (52.00, 360.50)	77.50 (40.00, 237.00)	96.00 (57.50, 531.00)	0.099
hs-CRP, mg/L	1.55 (0.61, 3.67)	1.97 (0.92, 3.90)	1.16 (0.55, 3.14)	0.084
CMR parameters				
LVEDV, mL	130.39 (97.40, 180.64)	122.20 (92.60, 179.82)	130.40 (106.00, 186.08)	0.507
LVESV, mL	69.30 (41.61, 121.57)	62.80 (36.70, 126.72)	76.10 (49.00, 118.30)	0.176
LVSV, mL	54.00 (38.86, 69.40)	56.30 (42.60, 72.50)	50.88 (36.20, 64.90)	0.269
LVEF, %	45.20 (25.79, 56.40)	51.70 (25.67, 60.40)	42.30 (26.80, 53.50)	0.095
LV mass, g	132.98 (99.45, 156.91)	132.82 (92.77, 163.99)	133.14 (111.48, 153.36)	0.564
LGE mass, g	25.32 (13.53, 46.09)	20.11 (6.74, 37.73)	31.98 (19.48, 53.00)	**0.001**
LGE %, %	20.70 (12.91, 31.06)	14.87 (6.87, 26.90)	23.78 (16.22, 33.65)	**< 0.001**

*LGE* late gadolinium enhancement. *LM* lipomatous metaplasia, *CCTA* coronary computed tomography angiography, *cTnI* cardiac troponin I, *CK-MB* creatine kinase, *BNP* B-type natriuretic peptide, *hs-CRP* high-sensitivity C-reactive protein, *LV* left ventricular, *EDV* left ventricular end diastolic volume, *LVESV* left ventricular end systolic volume, *LVSV* left ventricular stroke volume, *LVEF* left ventricular ejection fraction. Statistically significant *p*-values are in bold

**Fig. 6:**
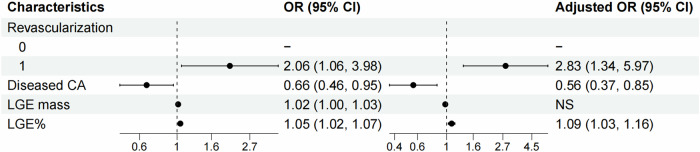
Forest plot of odds ratios for associations with LM occurrence in matched-pair analysis. Revascularization history and LGE% were directly associated with LM occurrence in univariable and multivariable logistic regression analysis, while the number of diseased coronary arteries was inversely associated with LM presence. OR, odds ratio; CA, coronary artery

Associations between LM presence and clinical and CMR parameters were evaluated in the matched-pair analysis (Supplementary Data, Table S5). Not all clinical parameters and CMR-based LV function parameters were significantly linked to LM. Revascularization history and LGE% were directly associated with LM presence in univariable (odds ratio, (OR) 2.056, 95% CI 1.063–3.976, *p* = 0.032; OR 1.046, 95% CI 1.018–1.074, *p* = 0.001; respectively) and multivariable (OR 2.833, 95% CI 1.343–5.972, *p* = 0.006; OR 1.094, 95% CI 1.031–1.160, *p* < 0.001; respectively) logistic regression analysis. The number of diseased coronary arteries was inversely associated with LM presence in both univariable (OR 0.660, 95% CI 0.458–0.951, *p* = 0.026) and multivariable (OR 0.556, 95% CI 0.366–0.846, *p* = 0.006) logistic regression analysis (see Fig. 6).

## Discussion

The prevalence, prognostic value and influencing factors of LM in patients after MI were investigated in this study. The following were the primary findings: (1) LM was a common complication following MI which most commonly occurs in myocardial infarctions older than 5 years, but could occur within 6 months; (2) LM was a significant outcome predictor in patients after MI, stronger than CMR functional parameters and infarct size; (3) revascularization history, infarct size and fewer number of diseased coronary arteries were associated with LM presence.

LM was initially reported to be a complication after MI in heart specimens after transplantation [18], with prevalence rates of 84% [1] and 68% [18] in histological studies and 11.43%–64% in noninvasive CT and MR studies [2, 10, 15, 19–21]. LM was reported to be significantly associated with infarct age, but was only found to be present in patients over 6 months post-MI in previous CT studies [2, 22]. In our study, LM was observed in 9 cases within 6 months after MI; one patient developed LM 2 months after MI. The discrepancy could be caused by small sample sizes of previous studies (less than 100 cases). Our results highlighted that LM was a common complication following MI and can occur shortly after the onset of MI. However, the clinical value of LM has not been adequately investigated.

Previous small sample studies focused on specific patients with MI. Cheniti et al examined 69 patients with post-myocardial infarction VT undergoing catheter ablation and reported that LM may be a potential marker of all-cause mortality and VT recurrence [21]. Kühl et al defined myocardial scar as myocardial fat and calcification detected on CCTA images. The scar was found to be an outcome predictor in patients with non-ST-segment elevation myocardial infarction [23]. Though a prior study reported that intramyocardial fat presence was independent of cardiovascular risks in the PROMISE study which analyzed 3705 patients with suspected stable CAD with no history of MI [24], the current study explored the prognostic value of LM detected on CCTA in a large cohort and found that LM on CCTA was a significant outcome predictor in patients after MI, the inconsistency could be due to differences in study cohorts. The prognostic significance and superiority of LM were further confirmed in the subgroup analysis. In the subgroup cohort, LM on CCTA was significantly associated with adverse outcomes; however, LM detected on CMR SSFP sequence showed no significant association. It is noteworthy that this is likely due to the smaller number of patients with LM detected on CMR compared to CCTA, reflecting that CCTA is much more sensitive in identifying LM than SSFP sequences, as small and sparse adipose tissue can be easily detected via attenuation thresholding on CCTA images, whereas it is not detectable on SSFP images, as the chemical shift effect requires a sufficient area of the high-signal island, visible width of the low-signal band, and adequate signal-nulling of the myocardium [6]. Another reason is that the spatial resolution of SSFP images is much lower than that of CCTA images, as CMR images are acquired with a larger slice thickness. The current result that LM on CMR was not a significant outcome predictor was inconsistent with a previous study [25]. The discrepancy may depend on different clinical outcomes and study cohorts; our study included a wider range of patients after MI and extensive adverse outcomes. The prognostic value of LM on CCTA was stronger than that of all CMR functional parameters and infarct size, and it remained significantly associated with poor outcomes when adjusted for all clinical and cardiac function parameters. This finding emphasizes the clinical significance of identifying LM on CCTA. Integration of LM assessment into risk stratification models for post-MI patients may be important for clinical strategy decisions.

The influencing factors of LM have been poorly understood and remain controversial in studies published so far. Su et al suggested that modern therapy for MI may promote LM [1], whereas Ichikawa et al did not find a significant difference in the prevalence of LM between reperfused and nonreperfused infarcts [2]. Lücke et al indicated that hyperlipoproteinemia may be a risk factor for LM development [10], but this finding has not been confirmed in other previous studies [3, 7, 20]. Moreover, all results of previous studies were not adjusted and could have been affected by confounding factors such as infarct age. Our study adjusted for infarct age and demographic factors in matched-pair analysis and found that revascularization history was linked to LM presence. The underlying reason may be that revascularization causes microvascular obstruction and intramyocardial hemorrhage in the reperfused postinfarct myocardium [26]. A recent study has observed that intramyocardial hemorrhage is the driving factor for LM formation in the infarct zone [27]. Myocardial scar size was found to be larger in the LM+ group than in the LM− group in previous studies [25, 28]. Nonetheless, their patient group was heterogeneous, and the relationship between the scar size and LM presence was not analyzed. As reported in a prospective CMR study conducted over a decade after MI, the evolution of myocardial scar size is a dynamic process, and the scar size tends to decrease progressively and significantly over time [29]. LM, on the contrary, tends to increase and progress over time, as noted in our study and previous investigations [2, 10]. The adipose tissue might be derived from fibrosis tissue in the extracellular space of the infarcted myocardium. In our study, the number of diseased coronary arteries was inversely associated with LM presence; it was consistent with a previous study [20]; however, this association was not found in another research [10]. The underlying mechanism needs further investigation.

The study has certain limitations. First, LM within the infarcted myocardium could not be pathologically confirmed. However, the CT threshold for fat attenuation was deemed to represent the adipose tissue. In addition, the chemical shift effect on SSFP sequences within the myocardial scar was proven to be fatty infiltration in a post-mortem case report [30]. Second, this study was performed retrospectively, and the causal relationship between clinical and imaging characteristics and LM after MI could not be determined. Some clinical information, such as time-to-revascularization and type of MI, may be associated with LM development, but was not analyzed due to a lack of related data. Hence, a prospective study is necessary for further investigation and validation. Finally, superior methods of CMR (like water-fat separation sequence) were not chosen for LM identification in this study, because certain sequences were not available due to the retrospective nature of the analysis.

## Conclusion

LM observed on CCTA images was a significant outcome predictor in patients after MI and was even stronger than CMR functional parameters and infarct size. Revascularization procedure, infarct size and fewer number of diseased coronary arteries may be associated with LM development.

## Supplementary information

ELECTRONIC SUPPLEMENTARY MATERIAL

## Data Availability

The datasets generated and/or analyzed during the current study are not publicly available due to patient privacy concerns. However, de-identified data may be made available from the corresponding author upon reasonable request, subject to approval by the relevant ethics committee.

## Abbreviations

CCTA: Coronary computed tomography angiography
CMR: Cardiovascular magnetic resonance
LGE: Late gadolinium enhancement
LM: Lipomatous metaplasia
MACE: Major adverse cardiac events
MI: Myocardial infarction
