# Supplementary material for: Prognostic significance and influencing factors of lipomatous metaplasia in patients after myocardial infarction
Source: Insights Imaging. 2025 Dec 8;16:270. doi: 10.1186/s13244-025-02152-w (PMC12686315; doi:10.1186/s13244-025-02152-w)
Supplement: Supplementary file 1 — ELECTRONIC SUPPLEMENTARY MATERIAL [file 13244_2025_2152_MOESM1_ESM.pdf]

Prognostic Significance and Influencing Factors of Lipomatous Metaplasia in Patients after Myocardial Infarction

ELECTRONIC SUPPLEMENTARY MATERIAL

Detailed CCTA acquisition protocols:

(1) Machine 1 (SOMATOM Force, Siemens): 2×96 detector, reconstructed slice thickness, 0.6mm; reconstructed slice interval, 0.5mm; tube voltage, 100 kVP; tube current, automated Smart-mA technique was used.

(2) Machine 2 (SOMATOM Definition Flash, Siemens): 2×64 detector, reconstructed slice thickness, 0.6mm or 0.75mm; reconstructed slice interval, 0.5mm; tube voltage, 100 kVP or 120 kVP; tube current, automated Smart-mA technique was used.

(3) Machine 3 (GE Revolution CT): 256 detector, reconstructed slice thickness, 0.6mm or 0.625mm; reconstructed slice interval, 0.5mm; tube voltage, 100 kVP; tube current, automated Smart-mA technique was used.

(4) Machine 4 (Aquilion ONE, TOSHIBA): 320 detector, reconstructed slice thickness, 0.5mm; reconstructed slice interval, 0.5mm; tube voltage, 100 kVP or 120 kVP; tube current, automated Smart-mA technique was used.

Detailed CMR acquisition protocols

CMR examinations were performed using three different 3.0 T systems: Achieva (Philips, the Netherlands), Magnetom Verio (Siemens, Erlangen, Germany), and Discovery MR750w (GE Healthcare, USA). All the three scanners are equipped with respiratory navigation electrocardiographic gating technology. 32-channel cardiac surface phased-array coil and parallel imaging technique were used. Cine and late gadolinium enhancement (LGE) images were used in this study. Cine images of 4-chamber view, 2-chamber view, 3-chamber view and short axis slices were acquired using SSFP sequences, LGE images of 4-chamber view, 2-chamber view and short axis slices were acquired using a phase-sensitive inversion recovery sequence.

(1) Machine 1 (Achieva, Philips, the Netherlands): 32-channel cardiac surface phased-array coil, parallel imaging technique was used, PE=2. FOV was adapted to the patient size. Mean RR was about 0.6-1.0s depending on the heart rate.

Cine imges: Balanced steady-state free precession (bSSFP) sequence. TR=2.9, TE=1.45, matrix=152 mm×150 mm, flip angle=45°, slice thickness=8-10 mm, gap=0 mm, bandwith=2284.1 Hz/pixel, number of heart beat was 5-10 per apnea depending on the heart rate. 30 phases per cardiac cycle. 25 views per segment.

LGE image: Phase-sensitive inversion recovery (PSIR) sequence. TR=6.1, TE=3.0, matrix=220 mm×160 mm, flip angle=25°, slice thickness=8-10 mm,

gap=0 mm, bandwith=228.2 Hz/pixel, number of heart beat was 6-12 per apnea depending on the heart rate. 30 views per segment.

(2) Machine 2 (Magnetom Verio, Siemens, Erlangen, Germany): 32-channel cardiac surface phased-array coil, parallel imaging technique was used, PE=2. FOV was adapted to the patient size. Mean RR was about 0.6-1.0s depending on the heart rate.

Cine images: True fast imaging with bSSFP sequence. TR=2.7, TE=1.35, matrix=256 mm×256 mm, flip angle=50°, slice thickness=8-10 mm, gap=0 mm, bandwith=814 Hz/pixel, 25 phases per cardiac cycle, number of heart beat was 5-10 per apnea depending on the heart rate. 30 views per segment.

LGE image: PSIR sequence. TR=5.4, TE=2.7, matrix=370 mm×350 mm, flip angle=20°, slice thickness=8-10 mm, gap=0 mm, bandwith=465 Hz/pixel, number of heart beat was 6-12 per apnea depending on the heart rate. 35 views per segment.

(3) Machine 3 (Discovery MR750w, GE Healthcare, USA): 32-channel cardiac surface phased-array coil, parallel imaging technique was used, PE=2. FOV was adapted to the patient size. Mean RR was about 0.6-1.0s depending on the heart rate.

Cine images: Fast imaging employing SSFP sequence. TR=3.6, TE=1.8, matrix=256 mm×192 mm, flip angle=60°, slice thickness=8-10 mm, gap=0 mm, bandwith=100 Hz/pixel, 25 phases per cardiac cycle, number of heart beat was 5-10 per apnea depending on the heart rate. 20-24 views per segment.

LGE images: PSIR sequence. TR=5.3, TE=2.5, matrix=220 mm×192 mm, flip angle=35°, slice thickness=8-10 mm, gap=0 mm, bandwith=41.67 Hz/pixel, number of heart beat was 6-12 per apnea depending on the heart rate. 24 views per segment.

TABLE S1 Intraobserver and interobserver agreement of LM on CCTA and LM on CMR

**Parameter**	**Intra-observer ICC (95% CI)**	**P value**	**Inter-observer ICC (95% CI)**	**P value**
LM on CCTA	0.978 (0.967, 0.985)	<0.001	0.887 (0.837, 0.923)	<0.001
LM on CMR	0.947 (0.923, 0.964)	<0.001	0.821 (0.734, 0.879)	<0.001

TABLE S2 Patient characteristics in the entire study cohort stratified by MACE occurrence

	All patients (n=1702)	MACE (-) (n=1307)	MACE (+) (n=395)	P value
LM on CCTA, n (%)	795 (46.71%)	577 (44.15%)	218 (55.19%)	**<0.001**
Age, years	59.3 ± 10.27	59.1 ± 10.36	59.9 ± 9.98	0.208
Sex, male, n (%)	1465 (86.08%)	1,127 (86.23%)	338 (85.57%)	0.740
Body mass index, kg/m^2	26.02 ± 3.28	26.04 ± 3.30	25.96 ± 3.22	0.671
Infarct age (years)	3.0 (1.0, 9.0)	3.0 (1.0, 8.0)	4.0 (1.3, 10.0)	**0.004**
Hypertension, n (%)	1058 (62.16%)	801 (61.29%)	257 (65.06%)	0.175
Diabetes mellitus, n (%)	637 (37.43%)	468 (35.81%)	169 (42.78%)	**0.012**
Hyperlipoidemia, n (%)	953 (55.99%)	721 (55.16%)	232 (58.73%)	0.210
Smoking, n (%)	1086 (63.81%)	829 (63.43%)	257 (65.06%)	0.553
Family history, n (%)	108 (6.35%)	74 (5.66%)	34 (8.61%)	**0.035**
Revascularization history, n (%)	1315 (77.26%)	998 (76.36%)	317 (80.25%)	0.106
PCI	981 (74.60%)	733 (73.45%)	248 (78.23%)	
CABG	184 (13.99%)	144 (14.43%)	40 (12.62%)	
PCI+CABG	150 (11.41%)	121 (12.12%)	29 (9.15%)	
Number of diseased coronary artery				0.166
No significant vessel stenosis, n (%)	3(0.18%)	3 (0.23%)	0 (0.00%)	
1-vessel disease, n (%)	431 (25.32%)	329 (25.17%)	102 (25.82%)	
2-vessel disease, n (%)	417 (24.50%)	320 (24.48%)	97 (24.56%)	
Multi-vessel disease (≥3), n (%)	851 (50.00%)	655 (50.11%)	196 (49.62%)	
Laboratory results				
cTNI, ng/mL	0.000 (0.000, 0.010)	0.000 (0.000, 0.010)	0.000 (0.000,	0.456

	All patients (n=1702)	MACE (-) (n=1307)	MACE (+) (n=395)	P value
			0.010)	
CK-MB, ng/mL	1.70 (1.20, 2.60)	1.70 (1.20, 2.50)	1.80 (1.10, 2.65)	0.336
BNP, pg/mL	80.00 (34.00, 190.01)	77.00 (33.00, 190.01)	89.00 (36.50, 190.01)	**0.029**
hs-CRP, mg/L	1.01 (0.48, 2.96)	0.98 (0.48, 2.84)	1.10 (0.49, 3.00)	0.300

Note: MACE: major adverse cardiac events; cTnI: cardiac troponin I; CK-MB: creatine kinase; BNP: B-type natriuretic peptide; hs-CRP: high-sensitivity c-reactive protein; CMR: cardiac magnetic resonance.

TABLE S3 Patient characteristics in subgroup analysis

	All patients (n=240)	MACE (-) (n=178)	MACE (+) (n=62)	P value
LM on CCTA, n (%)	123 (51.25%)	83 (46.63%)	40 (64.52%)	**0.015**
LM on CMR, n (%)	52 (21.67%)	36 (20.22%)	16 (25.81%)	0.308
Age, years	56.8 ± 10.90	57.0 ± 10.66	56.1 ± 11.64	0.612
Sex, male, n (%)	209 (87.08%)	156 (87.64%)	53 (85.48%)	0.663
Body mass index, kg/m^2	25.93 ± 3.716	26.13 ± 3.935	25.37 ± 2.956	0.114
Infarct age (years)	1.5 (0.5, 5.0)	1.5 (0.6, 5.0)	1.1 (0.3, 5.0)	0.594
Hypertension, n (%)	134 (55.83%)	99 (55.62%)	35 (56.45%)	0.909
Diabetes mellitus, n (%)	89 (37.08%)	67 (37.64%)	22 (35.48%)	0.762
Hyperlipoidemia, n (%)	122 (50.83%)	88 (49.44%)	34 (54.84%)	0.464
Smoking, n (%)	149 (62.08%)	112 (62.92%)	37 (59.68%)	0.650
Family history, n (%)	23 (9.58%)	13 (7.30%)	10 (16.13%)	**0.042**
Revascularization history, n (%)	108 (45.00%)	80 (44.94%)	28 (45.16%)	0.976
PCI	77 (71.30%)	53 (66.25%)	24 (85.72%)	
CABG	21 (19.44%)	18 (22.50%)	3 (10.71%)	
PCI+CABG	10 (9.26%)	9 (11.25%)	1 (3.57%)	
Number of diseased coronary artery				0.454
No significant vessel stenosis, n (%)	3 (1.25%)	3 (1.69%)	0 (0.00%)	
1-vessel disease, n (%)	78 (32.50%)	58 (32.58%)	20 (32.26%)	
2-vessel disease, n (%)	48 (20.00%)	39 (21.91%)	9 (14.52%)	
Multi-vessel disease (≥3), n (%)	111 (46.25%)	78 (43.82%)	33 (53.23%)	
Laboratory results				

	All patients (n=240)	MACE (-) (n=178)	MACE (+) (n=62)	P value
cTNI, ng/mL	0.010 (0.000, 0.070)	0.010 (0.000, 0.030)	0.020 (0.000, 0.220)	0.119
CK-MB, ng/mL	2.55 (1.60, 4.20)	2.30 (1.60, 3.70)	2.70 (1.63, 5.05)	0.131
BNP, pg/mL	85.50 (52.00, 418.25)	80.00 (52.00, 347.75)	106.50 (49.75, 747.00)	0.186
hs-CRP, mg/L	1.96 (0.69, 3.75)	1.85 (0.69, 3.68)	2.31 (0.80, 4.37)	0.270
CMR parameters				
LVEDV, ml	144.40 (105.43, 200.11)	136.28 (104.60, 186.04)	179.74 (109.63, 237.41)	**0.032**
LVESV, ml	82.67 (49.48, 150.63)	71.35 (46.82, 133.32)	119.90 (61.38, 183.71)	**0.007**
LVSV, ml	52.80 (38.15, 69.18)	54.54 (39.22, 70.56)	50.20 (36.02, 63.72)	0.295
LVEF, %	40.65 (24.15, 55.00)	43.66 (24.50, 55.90)	29.40 (22.29, 44.02)	**0.010**
LV mass, g	133.54 (103.96, 163.23)	130.55 (102.10, 161.00)	142.54 (112.10, 168.60)	0.187
LGE mass, g	29.51 (14.33, 53.42)	23.86 (10.63, 46.09)	42.51 (25.84, 63.31)	**<0.001**
LGE %	22.95 (12.87, 33.20)	19.11 (10.12, 28.61)	31.84 (21.97, 42.23)	**<0.001**

Note: LVEDV: left ventricular end diastolic volume; LVESV: left ventricular end systolic volume; LVSV: left ventricular stroke volume; LVEF: left ventricular ejection fraction; LGE: late gadolinium enhancement.

TABLE S4 Univariable and multivariable Cox regression results for MACE association in subgroup analysis

	Univariable analysis		Multivariable analysis	
	**Hazard Ratio (95% CI)**	**P value**	**Hazard Ratio (95% CI)**	**P value**
LM on CCTA, n (%)	1.83 (1.09, 3.08)	**0.023**	1.45 (0.84, 2.50)	0.185
LM on CMR, n (%)	1.46 (0.82, 2.59)	0.194		
Age, years	1.00 (0.98, 1.02)	0.893		
Sex, male, n (%)	1.29 (0.63, 2.62)	0.484		
Body mass index, kg/m^2	0.98 (0.91, 1.05)	0.560		
Infarct age (years)	1.00 (1.00, 1.01)	0.315		
Hypertension, n (%)	0.94 (0.56, 1.56)	0.807		
Diabetes mellitus, n (%)	0.95 (0.56, 1.60)	0.848		
Hyperlipoidemia, n (%)	1.54 (0.93, 2.56)	0.096		
Smoking, n (%)	1.09 (0.65, 1.81)	0.750		
Family history, n (%)	2.39 (1.20, 4.75)	**0.013**	3.06 (1.47, 6.35)	**0.003**
Revascularization history, n (%)	1.01 (0.61, 1.67)	0.959		
Number of diseased coronary artery	1.10 (0.83, 1.44)	0.506		
**Laboratory results**				
cTNI, ng/mL	1.00 (0.99, 1.00)	0.827		
CK-MB, ng/mL	1.00 (0.99, 1.02)	0.872		
BNP, pg/mL	1.00 (1.00, 1.00)	**0.022**	1.00 (1.00, 1.00)	0.515
hs-CRP, mg/L	1.01 (1.01, 1.03)	0.126		
**CMR parameters**				
LVEDV, ml	1.01 (1.00, 1.01)	**<0.001**	1.01 (0.99, 1.03)	0.318
LVESV, ml	1.01 (1.00, 1.01)	**<0.001**	1.00 (0.97, 1.02)	0.754

	Univariable analysis		Multivariable analysis	
	**Hazard Ratio (95% CI)**	**P value**	**Hazard Ratio (95% CI)**	**P value**
LVSV, ml	1.00 (0.99, 1.02)	0.509		
LVEF, %	0.98 (0.97, 0.99)	**0.004**	1.01 (0.96, 1.06)	0.690
LV mass, g	1.01 (1.00, 1.01)	**0.041**	1.00 (0.99, 1.01)	0.988
LGE mass, g	1.02 (1.01, 1.03)	**<0.001**	1.00 (0.96, 1.03)	0.849
LGE %	1.04 (1.02, 1.05)	**<0.001**	1.00 (0.98, 1.10)	0.235

Note: 95% CI: 95% confidence interval; LM: lipomatous metaplasia; CCTA: coronary computed tomography angiography.

TABLE S5 Univariable and multivariable logistic regression analysis for LM association in matched-pair patients

	Univariable analysis		Multivariable analysis	
	**Odds Ratio (95% CI)**	**P value**	**Odds Ratio (95% CI)**	**P value**
**Demographic characteristics**				
Age, years	0.987 (0.957, 1.019)	0.422		
Sex, male, n (%)	1.000 (0.373, 2.683)	1.000		
Body mass index, kg/m^2	1.071 (0.968, 1.184)	0.183		
Infarct age (months)	1.001 (0.994, 1.008)	0.826		
**Cardiovascular risk factors**				
Hypertension, n (%)	0.747 (0.381, 1.461)	0.394		
Diabetes mellitus, n (%)	0.679 (0.353, 1.306)	0.246		
Hyperlipoidemia, n (%)	1.116 (0.583, 2.136)	0.741		
Smoking, n (%)	0.747 (0.381, 1.461)	0.394		
Family history, n (%)	0.821 (0.239, 2.820)	0.754		
Revascularization history, n (%)	2.056 (1.063, 3.976)	**0.032**	2.833 (1.343, 5.972)	**0.006**
Number of diseased coronary artery	0.660 (0.458, 0.951)	**0.026**	0.556 (0.366, 0.846)	**0.006**
**Laboratory results**				
cTNI, ng/mL	1.000 (1.000, 1.001)	0.540		
CK-MB, ng/mL	0.996 (0.982, 1.010)	0.547		
BNP, pg/mL	1.000 (1.000, 1.000)	0.504		
hs-CRP, mg/L	0.962 (0.919, 1.006)	0.089		
**CMR parameters**				
LVEDV, ml	1.000 (0.996, 1.005)	0.821		

	Univariable analysis		Multivariable analysis	
	**Odds Ratio (95% CI)**	**P value**	**Odds Ratio (95% CI)**	**P value**
LVESV, ml	1.001 (0.996, 1.005)	0.719		
LVSV, ml	0.996 (0.981, 1.011)	0.611		
LVEF, %	0.986 (0.969, 1.004)	0.132		
LV mass, g	1.001 (0.994, 1.008)	0.765		
LGE mass, g	1.015 (1.002, 1.029)	**0.028**	0.978 (0.951, 1.007)	0.130
LGE %	1.046 (1.018, 1.074)	**0.001**	1.094 (1.031, 1.160)	**0.003**
